# The model of cytokine release syndrome in CAR T-cell treatment for B-cell non-Hodgkin lymphoma

**DOI:** 10.1038/s41392-020-00256-x

**Published:** 2020-07-29

**Authors:** Jianshu Wei, Yang Liu, Chunmeng Wang, Yajing Zhang, Chuan Tong, Guanghai Dai, Wei Wang, John E. J. Rasko, J. Joseph Melenhorst, Wenbin Qian, Aibin Liang, Weidong Han

**Affiliations:** 1 Department of Bio-therapeutic, the First Medical Center, Chinese PLA General Hospital, 100853 Beijing, China; 2 Department of Oncology, the first Medical Center, Chinese PLA General Hospital, 100853 Beijing, China; 3 State Key Laboratory of Biotherapy and Cancer Center, West China Hospital, Sichuan University, Chengdu, 610041 China; 4 Collaborative Innovation Center for Biotherapy, Chengdu, 610041 China; 5 Department of Cell and Molecular Therapies, Royal Prince Alfred Hospital, Sydney, NSW 2006 Australia; 6 Gene and Stem Cell Therapy Program, Centenary Institute, University of Sydney, Sydney, NSW 2006 Australia; 7 Central Clinical School, Faculty of Medicine and Health, The University of Sydney, Camperdown, NSW 2050 Australia; 8 Department of Pathology and Laboratory Medicine, Perelman School of Medicine, University of Pennsylvania, Philadelphia, PA 19104-515 USA; 9 Department of Hematology, The Second Affiliated Hospital, College of Medicine, Zhejiang University, Hangzhou, 310009 China; 10 Department of Hematology, Shanghai Tongji Hospital, Tongji University School of Medicine, Shanghai, 200065 China; 11 State Key Laboratory of Kidney Diseases, National Clinical Research Center for Kidney Diseases, 100853 Beijing, China

**Keywords:** Immunotherapy, Inflammation

## Abstract

Chimeric antigen receptor T (CAR T) cell therapy has demonstrated efficacy in the treatment of haematologic malignancies. However, the accompanying adverse events, the most common of which is cytokine release syndrome (CRS), substantially limit its wide application. Due to its unique physiological characteristics, CRS in CAR T-cell treatment for B-cell non-Hodgkin lymphoma (B-NHL) may exhibit some special features. Although existing guidelines had greatly promoted the recognition and management of CRS, many recommendations are not fully applicable to B-NHL. Therefore, it is imperative to identify responses that are specific to CRS observed following CAR T treatment for B-NHL. Based on underlying biological processes and known pathophysiological mechanisms, we tentatively propose a new model to illustrate the occurrence and evolution of CAR T-cell-therapy-related CRS in B-NHL. In this model, tumour burden and bone marrow suppression are considered determinants of CRS. Novel phenomena after CAR T-cell infusion (such as local inflammatory response) are further identified. The proposed model will help us better understand the basic biology of CRS and recognize and manage it more rationally.

## Introduction

Chimeric antigen receptor T (CAR T) cell therapy has emerged as a promising therapeutic approach for haematological malignancies,^1–6^ and overall response rates of 52–82% and durable remission could be achieved in patients with refractory or relapsed (R/R) B-cell malignancies.^5,7–9^ Consequently, two CAR T-cell products targeting CD19 have been approved for R/R B-cell acute lymphoblastic leukaemia (B-ALL) and B-cell non-Hodgkin lymphoma (B-NHL) by the U.S. Food and Drug Administration.^10,11^

In vivo, infused CAR T cells will specifically recognize and eliminate tumour cells expressing the target antigen. At the same time, these CAR T cells, activated by CAR mediated signals, will proliferate and release a variety of inflammatory factors to trigger a systemic inflammatory response.^12,13^ Therefore, CAR T-cell therapy often produces significant adverse events (AEs), with the most common being cytokine release syndrome (CRS).^5,7,9,14^ Progressive CRS can cause serious morbidity in patients or reduce the clinical benefit due to the use of measures intended to control CRS, such as corticosteroids.^15,16^ Therefore, proper recognition and management of CRS may not only alleviate toxicity but also improve the likelihood of therapeutic benefit.

**Fig. 1:**
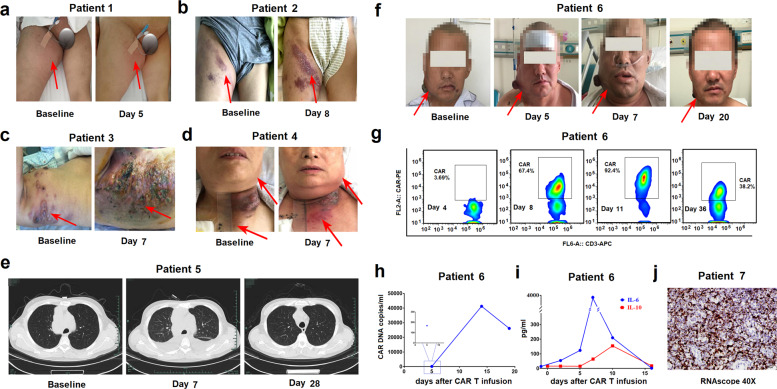
Clinical manifestations of local cytokine release syndrome (L-CRS). **a**–**f** In the early stage of CAR T-cell treatment, B-NHL patients may exhibit a significant local inflammatory response, mainly manifested as local swelling and redness. For example, patient 6 had a significant L-CRS response within 5 days after receiving CAR T treatment, but during this period, the proportion of CAR T cells to CD3 positive cells (**g**), the number of CAR DNA copies in PB (**h**) and the level of IL-6 in PB (**i**) remained at a low level. **j** The RNAscope results of patient 7 indicated that a large number of CAR T cells infiltrated into B-NHL lesions. B-NHL B-cell non-Hodgkin lymphoma, CAR chimeric antigen receptor, IL-6 interleukin-6, PB peripheral blood

B-NHL has distinct pathophysiological characteristics and clinical manifestations from other haematological malignancies, such as B-ALL. An obvious point of difference is that the lesions of B-NHL are generally localized. Given the nature of CAR T cells to pursue target cells, the in vivo dynamics of CAR T cells in B-NHL could be fundamentally different from those in other cancers. Consequently, the related toxicities could also exhibit unique features. For example, in B-NHL patients receiving CAR T-cell therapy, compartmental inflammation can be observed, manifested as redness, swelling and enlargement at the local lymphoma or around the periphery of lesions (Fig. 1). Parallel local inflammatory reactions have also been reported in several other reports.^17–19^ To draw a contrast to the widely recognized systemic CRS (S-CRS), we tentatively define this local inflammatory response as local CRS (L-CRS).

**Fig. 2:**
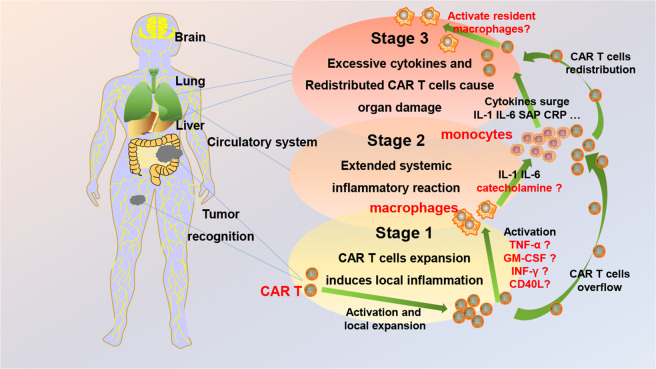
A new model to illustrate the occurrence and evolution of chimeric antigen receptor T (CAR T) cell therapy-related adverse events (AEs) in B-cell non-Hodgkin lymphoma (B-NHL). Stage 1: CAR T cells converge upon tumour cells and kill them. The early distribution of CAR T cells is localized, and the activated CAR T cells release cytokines that in turn trigger a series of local inflammatory reactions, defined in this paper as local cytokine release syndrome (L-CRS). Stage 2: Locally elevated CAR T-cell numbers and cytokine ‘overflow’ into the circulatory system occur, which may boost systemic cytokine release syndrome (S-CRS). Stage 3: CAR T cells redistribute into bone marrow and normal organs, such as the liver, lung and brain. The redistributed CAR T cells might activate tissue-resident immune cells to cause local organ damage and other AEs. TNF tumour necrosis factor, GM-CSF granulocyte-macrophage colony-stimulating factor, IFN interferon, CCL-3 C-C motif chemokine ligand 3 (also known as macrophage inflammatory protein 1α, MIP-1α), IL interleukin, SAP serum amyloid P component, CRP C-reactive protein, BM bone marrow

Currently, our understanding of B-NHL-specific AEs induced by CAR T-cell therapy is still lacking, and published CRS grading and management guidelines give little specific recommendations for B-NHL.^15,20,21^ To better guide clinical work and basic research, we tentatively propose a new model to illustrate the occurrence and progression of CRS for B-NHL based on existing clues and our practical clinical experience (Fig. 2). As shown in Table 1, the progression of CRS is divided into four stages: (1) CAR T-cell local expansion stage; (2) CAR T-cell overflow and inflammatory cytokine surge stage; (3) CAR T-cell redistribution and organ damage stage and 4 recovery stage (or immune reconstruction). In this model, tumour burden and bone marrow suppression (BMS) are considered to be the determinants of CRS. Besides, we review and describe our recognition and clinical management of CRS in CAR T treatment for B-NHL, as well as our perspectives on the underlying mechanisms.

**Table 1:** Four different stages of the occurrence and progress of CRS in CAR T-cell treatment for B-NHL

	Stage 1 “Expansion”	Stage 2 “Cytokine surge”	Stage 3 “Redistribution”	Stage 4 “Recovery”
CAR T kinetics	CAR T cells in PB increase mildly. Percentage of CAR^+^ in CD3^+^ T: 0–20%.	CAR T cells in PB increase rapidly until the peak. Percentage of CAR^+^ in CD3^+^ T: 20–95%.	CAR T cells in PB begin to decline. Percentage of CAR^+^ in CD3^+^ T: 30–50%.	CAR T cells gradually decrease to a very low level. Percentage of CAR^+^ in CD3^+^ T: <20%.
IL-6 in PB	IL-6 increases mildly, sometimes with a minimal peak. Against baseline: 1–5 times.	IL-6 increases rapidly until the peak. Against baseline: >20 times.	IL-6 gradually declines, sometimes with a secondary peak. Against baseline: 2–20 times.	IL-6 continues to decline to normal. Against baseline: 1–3 times.
Lesions	Significant swelling and redness. Against baseline: 1–1.2 times (fold change in diameter).	Swelling continues for a period of time, and then the tumours begin to shrink. Against baseline: 0.2–1.5 times (fold change in diameter).	The tumours continue to shrink. Against baseline: 0.1–1.2 times (fold change in diameter).	The tumours disappear or relapse.
WBC	Continuous decline. Myelosuppression: II–IV.	WBC count gradually increase, accompanied by agranulocytosis. Myelosuppression: III–IV.	After a minimal descending, WBC count gradually increase. Myelosuppression: II–III.	WBC count gets to normal levels. Myelosuppression: II–III.
ALT/AST	Little change can be observed.	ALT/AST rapidly rises to peak. Against baseline: 2–5 times.	A transient rise of ALT/AST. Against baseline: 1–3 times.	ALT/AST gradually gets to normal. Against baseline: 1–1.5 times.
B-NHL				
		ALL		

Stage 1. Local CAR T cells expansion stage; Stage 2. CAR T cells overflow and inflammatory cytokines surge stage; Stage 3. CAR T cells redistribution stage; Stage 4. Recovery (immune reconstruction) stage

*CAR* chimeric antigen receptor, *IL-6* interleukin-6, *PB* peripheral blood, *WBC* white blood cell, *ALT/AST* alanine aminotransferase/aspartate aminotransferase, *B-NHL* B-cell non-Hodgkin lymphoma, *ALL* acute lymphoblastic leukaemia

## Patterns of progression in CRS

After infusion, CAR T cells will rapidly locate and gather around tumour cells in a short time to kill them via contact-dependent cytotoxicity.^13,22,23^ Therefore, the early distribution of CAR T cells should be mostly localized to compartments containing B-NHL lesions. Current studies have demonstrated that activated monocytes and macrophages are major contributors to the “amplification” of the inflammatory response^13,24^ in CAR T-cell therapy. For the activation of monocytes/macrophages, the direct contact between CAR T cells and them is considered to play an important role,^25,26^ even more important than cytokines.^27,28^ For example, CD40-CD40L,^29,30^ CD69,^31^ lymphocyte activation gene-3^32^ and membrane expressed TNF-α^33,34^ have been demonstrated to activate monocytes/macrophages through contact-dependent mechanisms. Consequently, the activation of monocytes/macrophages, as well as the progression of CRS, should be related to the in vivo distribution of CAR T cells.

We therefore argue that the CRS in B-NHL patients should exhibit different patterns of progression due to the unique in vivo dynamics of CAR T cells. A goal of our studies was to understand the characteristics of CRS progression in B-NHL patients receiving CAR T-cell treatment to better guide clinical management and basic research. Here, we tentatively propose a new model to illustrate the occurrence and progression of CRS in B-NHL based on existing clues and our practical clinical experience administering CAR T-cell treatment for B-NHL patients. In this model, we have defined four distinct stages (Table 1).

**Fig. 3:**
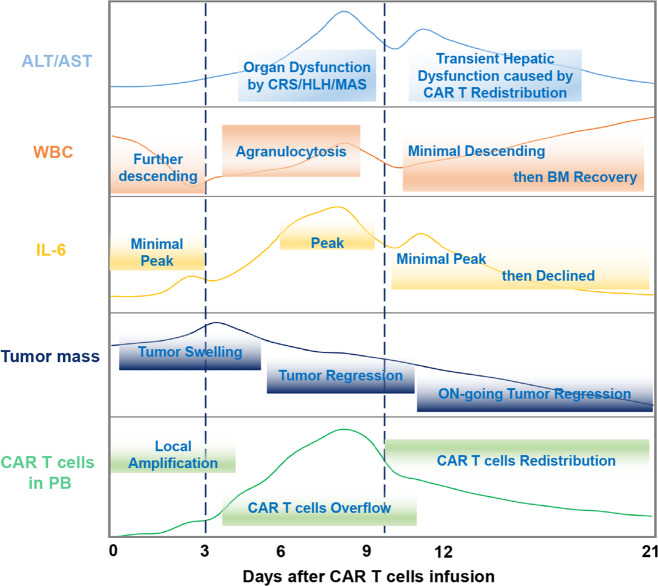
The in vivo kinetics of chimeric antigen receptor T (CAR T) cells and associated events in the early stage of CAR T-cell therapy for B-cell non-Hodgkin lymphoma (B-NHL). Within ~3 days after infusion, CAR T cells proliferate locally in the tumour, and the number of CAR T cells in the peripheral blood (PB) increases slowly, accompanied by enlargement of tumour lesions, a mild rise in IL-6 in the PB and an initial minimal peak and further decline of white blood cell (WBC) counts caused by preconditioning chemotherapy. Within ~3–10 days after infusion, a large number of CAR T cells overflow from the tumour site into the PB, accompanied by obvious regression of tumours, a rapid rise in IL-6 in PB to a peak (of note, the peak level of IL-6 is generally seen 1–2 days earlier than that of CAR T-cell numbers), a slow rise in WBC count (due to the accumulation of CAR T cells in PB) and agranulocytosis and organ damage caused by cytokine release syndrome (CRS) or haemophagocytic lymphohistiocytosis/macrophage activation syndrome (HLH/MAS). Within about 10–21 days after infusion, the peripheral CAR T cells redistribute into BM and normal organs (prompted by a rapid decrease in CAR T cells in PB), accompanied by ongoing tumour regression, a minimal peak and then continuous decline in IL-6 levels (possibly because the redistributed CAR T cells activate monocytes/macrophages in the BM and normal tissues) and WBC count recovery after a minimal dip accompanied by transient hepatic dysfunction. IL-6 interleukin-6, AST aspartate aminotransferase, ALT alanine aminotransferase, BM bone marrow

In the first stage, infused CAR T cells aggregate in tumour masses and expand locally. This stage is usually observed 0–5 days after CAR T infusion. During this period, sustained intra-tumoral expansion of CAR T cells can be retained within the tumour mass, and few CAR T cells recirculate into the peripheral blood (PB) (Fig. 3).^17^ At the same time, activated CAR T cells release a large number of cytokines, by which a local inflammatory response is triggered. Tumour-infiltrating macrophages and dendritic cells may enhance local inflammation, although it is not clear how important their roles are and how they are activated. During this period, many local inflammatory manifestations can be observed clinically.^35^

In the second stage, locally expanded CAR T cells and cytokines begin to significantly enter the circulatory system. This stage usually occurs 3–12 days after CAR T-cell infusion according to our clinical observations. Early in this stage, a rapid increase in CAR T cells and inflammatory factors (such as IL-6) in PB can be observed (Fig. 3). In the following week or two, the levels of CAR T cells and inflammatory factors in PB will continue to rise until the peak. Usually, the most intense S-CRS occurs at this stage. The underlying mechanisms of CRS at this stage are likely similar to those of the CRS observed with CAR T-cell treatment for ALL. During this period, monocytes that are eliminated by preconditioning begin to recover gradually.^36,37^ Therefore, newborn monocytes in the circulatory system and bone marrow (BM) could be another contributor to S-CRS.^13,24,38–41^ The main clinical manifestations of this stage include intractable fever, decreased blood pressure, impaired lung function, liver damage, increased exudation in the serosal cavities, abnormal blood coagulation, BMS and so on.^15,42^ It should be noted that the first and second stages are sometimes blended in practice, especially when a patient has very large tumour masses or an inflammatory background (such as infection).^43^ Generally, serious systemic inflammatory responses start earlier than mild responses after CAR T-cell infusion.^12^ If not handled properly, they can lead to high mortality.

In the third stage, the proliferation of CAR T cells is retarded owing to the lack of antigen stimulation, leading to a lower number of CAR T cells in PB. This stage usually occurs 10–21 days after CAR T-cell infusion.^5,9,44^ Early in this stage, a small decrease of white blood cells in the PB can also be observed (according to our clinical observations). Meanwhile, a relatively rapid decrease in the number of peripheral CAR T cells and liver damage can be observed (Fig. 3).^2,45^ These clues suggest the redistribution of CAR T cells, which might be attributed to the absence of target cells.^22,46^ The redistributed CAR T cells might activate tissue-resident immune cells such as macrophages or neutrophils to cause organ damage and other AEs.^12,47–50^ In this process, cytokines that diffuse from PB into organs are also believed to play important roles, such as IL-6, CRP and fibrinogen.^12,51–54^ The main manifestations of this period include secondary organ damage and a secondary cytokine peak (involving cytokines such as IL-6).

If CRS could be effectively controlled, then the fourth stage (recovery stage) occurs. For the recovery, continuous tumour regression and retarded CAR T-cell proliferation are the prerequisite requirements. During this period, the number of CAR T cells in PB continues to decline, and inflammatory factors return towards normal levels (Fig. 3). The BMS is gradually relieved and haematopoiesis begins to resume, accompanied by a gradual increase in the peripheral leucocyte count. Regulatory immune cells, such as myeloid-derived suppressor cells (MDSCs) and regulatory T cells (T-regs), are also replenished, which should be an important factor preventing secondary expansion of CAR T cells.^55–57^ This stage usually begins ~3 weeks after the CAR T-cell infusion.

These four stages are distinguished to illustrate the different phases of CRS based on the in vivo kinetics of CAR T cells. Although the mechanisms underlying S-CRS should be similar following treatment with CAR T cell in other malignancies, the occurrence of L-CRS would be relatively specific to B-NHL. The local inflammatory response (leading to L-CRS) produces a large number of activated CAR T cells and cytokines, which are released into the PB to trigger a systemic inflammatory response. Therefore, L-CRS can be regarded as the “trigger” of subsequent S-CRS, and timely recognition and management of it are necessary in order to reduce the harm of subsequent S-CRS.

## Determinants of CRS

The intensity and kinetics of CAR T-cell-therapy-related CRS significantly vary among different patients. The accumulation of clinical cases suggests that CRS could be affected by a combination of factors, including tumour burden, individual immune status, the peak number of CAR T cells, IL-6 level, lymphodepletion and the gut flora.^9,12,37,58–63^ Clarifying how these factors interact will help us better understand the general progression pattern of CRS and thus more effectively predict and manage the related toxicity.^41^

In principle, CAR T cells and their released cytokines coordinate in generating CRS. Therefore, the in vivo expansion of CAR T cells driven by target cell-mediated stimulation should be one of the core determinants.^41^ Generally, the higher the levels of CAR T cells are, the higher the degree of toxicity observed.^2^ Of course, CAR T-cell amplification is also positively correlated with clinical benefits.^8,45^ Tumour burden not only determines the peak value of CAR T cells but also may affect the time course of CAR T-cell proliferation. For most patients, a rapid rise in inflammatory factors occurs within 5–10 days after CAR T-cell transfusion.^9,64^ After the peak, CAR T-cell amplification will slow down and the level of cytokines will decrease as the tumour burden subsides. However, if the tumour has not been eliminated substantially over a period of time (e.g., 1 month), the CRS might be prolonged. In practice, we also found that B-NHL patients with large tumour burden were more likely to experience severe L-CRS and S-CRS than those with a small tumour burden, and high-level CRS has been reported to have a longer duration than mild CRS.^12^

In addition to the tumour burden, another decisive factor of CRS intensity is the level to which haematopoiesis is suppressed. BMS is first caused by preconditioning regimens, of which the fludarabine/cyclophosphamide (FC) lymphodepletion regimen is the most widely used to improve therapeutic efficacy.^65–67^ Lymphodepletion can reduce the competition of other lymphocytes for growth factors, provide more ‘space’ for CAR T-cell proliferation and remove the inhibitory immune cells such as T-regs and MDSCs,^68^ thus promoting in vivo CAR T-cell expansion.^69–71^ During the BMS period, the expansion of CAR T cells is barely restricted, thereby facilitating target cell-mediated stimulation, a consequent peak of CAR T cells and acute systemic inflammatory response. Generally, more than 3 weeks are needed for the impaired haematopoietic function to return to normal after FC precondition.^36^ After this, CAR T-cell proliferation will be gradually limited along with the recovery of haematopoiesis, and CRS will also be alleviated.

This is the common progression pattern of BMS and CRS, but sometimes the BM recovery does not proceed efficiently,^8,71,72^ and it is even further aggravated by activated CAR T cells that redistribute into the BM.^41^ Poor haematopoiesis may increase the risk of secondary active proliferation of CAR T cells, especially for patients with high tumour burden. In addition, the persistent impaired haematopoietic function also increases the risk of infection,^73–75^ which is sometimes life-threatening. However, the mechanisms by which redistributed CAR T cells aggravate BMS remain to be further investigated.

The dose of administrated CAR T cells has also been considered a key factor of determining the intensity and kinetics of CRS by other scientists.^41,76^ Reducing the CAR T dose is considered a possible way to reduce CRS, as has been tested by several trials.^77,78^ Although there are some supporting data, the main concern is that the dose reduction will impair the therapeutic benefits, which is particularly concerning in B-NHL.^51^ Therefore, until clear evidence is provided, we do not recommend reducing the CAR T-cell dose to reduce the severity of CRS.

## Recognition and clinical management of AEs

### Local CRS

L-CRS can only occur in compartmental tumours and has only been reported in a few papers so far.^17–19^ In the case of solid tumours, the lack of examples is mainly due to the low effectiveness of CAR T-cell treatments.^56,79^ Heterogeneity and the immunosuppressive microenvironment are viewed as the main factors limiting the efficacy of CAR T-cell treatments for solid tumours.^55,80^ In addition, the infiltration of CAR T cells in the tumour bed could be further reduced by physical barriers.^81^

For B-NHL, significant L-CRS is also not frequently observed, mainly because patients with large masses are usually excluded or they are pretreated with chemotherapy to reduce the tumour burden before CAR T-cell treatment. In contrast, significant L-CRS was more frequently observed in our clinical trials for patients with large tumour masses. As shown in Fig. 1, several patients with B-NHL had a significant inflammatory response in the tumour site in the early stage after CAR T-cell infusion. The L-CRS is mainly affected by tumour burden and the consequent CAR T-cell amplification, and it usually occurs within 0–5 days after CAR T-cell infusion. During this period, S-CRS can also occur. However, the low number of CAR T cells and IL-6 levels in PB are in stark contrast to the conspicuous local inflammatory response (Fig. 1).^17^

L-CRS is the earliest AE observed following CAR T-cell treatment for B-NHL. Local proliferation serves as a reservoir of the continuous release of activated CAR T cells and cytokines that induce the systemic immune response. Therefore, timely control of L-CRS is helpful to prevent severe S-CRS. However, in early clinical practice, we found that IL-6 blocking antibodies could not effectively alleviate and instead aggravated the L-CRS response. This indicated that the underlying mechanisms of L-CRS might be specific.

It remains unclear which types of immune cells take part in this process and how these immune cells are activated. Tracking different infiltrating lymphocytes to clarify their changes in distribution and function may provide further insights into the pathogenesis of L-CRS. For this, a proper animal model for studying L-CRS is imperative. Considering the potentially important role of macrophage activation in the progression of L-CRS and S-CRS, we have previously attempted to control the inflammatory response by inhibiting macrophage activation using TNF-a blocking drugs^82^ in the patients with a high tumour burden (SPD ≥ 100 cm^2^).

For patients without compression symptoms, continuous clinical observation and supportive care are recommended. When compression symptoms occur, anti-TNF-α therapy and local intervention (if necessary), such as tracheotomy and drainage of serous effusion, should be implemented.

The specific damage caused by L-CRS can vary with the location of lesions. Of note are the masses located in or around the intestine. The local inflammatory response can cause damage to the intestines, such as intestinal mucosal damage, intestinal vascular rupture and bleeding. Such tissue damages provide more possibilities for the intestinal flora to boost the systemic immune response.^62,83–86^ For patients with tumours in proximity to the intestines, in particular with large tumours, gut purging before CAR T-cell treatment and oral antibiotics to inhibit the intestinal flora are recommended. The intestinal flora will be reconstructed after the S-CRS subsides. Recently, it was reported that antibiotic therapy may reduce the effectiveness of immunotherapy,^87,88^ whether the anti-tumour potential of CAR T cells will be weakened by the use of antibiotics needs further study. For these patients, prophylactic use of anti-TNF-α agents can also be considered. Another organ that needs attention is the heart, and more careful monitoring of cardiac function should be given when the B-NHL mass is located around the heart.

### Systemic CRS

S-CRS, the most common AE associated with CAR T-cell treatment, is characterized by fever, hypotension, hypoxia and increased release of inflammatory cytokines,^89,90^ including IL-1, IL-6, IFN-γ, TNF-α, GM-CSF, MIP-1α, MCP1 and IL-10. S-CRS is commonly reversible following medical intervention. But severe S-CRS can lead to multiple organ dysfunctions, such as persistent cytopenias, cardiac complications, acute kidney injury and coagulation system abnormalities. According to published data, any grade CRS occurred in 42–93% of B-NHL patients receiving CAR T-cell treatment, with grade ≥ 3 toxicity occurring in 2–22% of patients.^7,9,72^

As discussed, the kinetics of S-CRS is closely related to tumour burden. Significant S-CRS generally occurs during the period when the number of CAR T cells increases in the PB, which usually peaks within 2 weeks after infusion. After this, the CRS response usually subsides due to the slowing down of CAR T-cell proliferation. However, sometimes S-CRS may persist for a longer time due to the continuous expansion of CAR T cells, which occurs if the residual tumour remains substantial. Due to the recovery of the haematopoietic function of the BM, some immunosuppressive cells can be reconstituted.^68,91^ Therefore, prolonged S-CRS is generally mild, characterized by persistent manifestations of low-level inflammation. For patients with incomplete BM recovery, chronic CRS can ignite secondary acute CRS under certain conditions, which is clinically dangerous.

As the most common AE, S-CRS can give rise to other CAR T-cell-therapy-related AEs, such as immune effector cell-associated neurotoxicity syndrome and haemophagocytic lymphohistiocytosis.^67^ Therefore, the recognition and management of S-CRS are very important issues, which have been widely reviewed. And some consensus guidelines have also been developed.^15,16,21,92^

In early CAR T clinical trials, the Common Terminology Criteria for Adverse Events (CTCAE) v4.03 released in 2010 was used for CRS grading. However, the criteria were mainly for antibody drugs, which did not reflect the dynamics of CRS induced by CAR T-cell therapy. In 2014, Lee et al. proposed their CRS grading system. Specific clinical indicators such as patient response to vasopressors, oxygen requirement and organ toxicity were included in the criteria. In CTCAE v5.0 released in 2018, many criteria from the Lee grading system were used for reference. In 2018, the CRS grading system of the University of Pennsylvania was proposed. The greatest controversy over this grading system over other guidelines is that any fluid bolus or vasopressor use is indicative of CRS grade 3, and this is believed to improperly increase the proportion of high-level CRS. In the same year, Neelapu et al. published the CAR T‑cell‑therapy‑associated toxicity (CARTOX) grading system, which is very similar to the Lee’s guidelines. In CARTOX, body temperature, blood pressure, oxygen saturation and organ damage are used for CRS grading. In 2019, the American Society for Transplantation and Cellular Therapy released their consensus recommendations on CRS grading. Compared with CARTOX, these recommendations held that fever ≥ 38 °C was necessary for CRS recognition, and organ damage was removed from the grading system. Use of one or more vasopressors was employed as an indicator for CRS grading instead of the dose of vasopressor. In addition, consideration of the modality of oxygen delivery instead of just FiO_2_ was adopted.

Our recognition and management of S-CRS in CAR T-cell treatment for B-NHL are presented in Table 2, which are generally consistent with the CARTOX criteria.^15^ Briefly, S-CRS can be effectively controlled below grade 3 by the use of tocilizumab in most cases. For cases of S-CRS refractory to tocilizumab therapy, corticosteroid therapy is recommended when the grade ≥ 3 CRS is observed. Of note, corticosteroids have been shown to significantly inhibit T-cell activity, leading to impaired clinical outcomes.^20,52^ When CRS reaches grade ≥ 4, more intense treatments, such as methylprednisolone and mechanical ventilation, should be provided in a timely manner to prevent severe morbidity and death.^93^

**Table 2:** The recognition and clinical management of CRS in CAR T-cell treatment for B-NHL

	CARTOX grading and management	CRS grading and management for B-NHL	
	CRS	L-CRS	S-CRS
Occurrence time	Day 1–14	Day 1–10	Day 2–14
Grading	Grade 1: (1) Temperature ≥ 38 °C. (2) Grade 1 organ toxicity.	Low-risk: (1) Diameter max < 10 cm. (2) No risk of tumour compression.	Grade 1: Consistent with CARTOX.
	Grade 2: (1) Hypotension responds to IV fluids or low-dose vasopressors. (2) Hypoxia requiring FiO_2_ < 40% (3) Grade 2 organ toxicity.		Grade 2: Consistent with CARTOX.
	Grade 3: (1) Hypotension needing high-dose or multiple vasopressor. (2) Hypoxia requiring FiO_2_ ≥ 40%. (3) Grade 3 organ toxicity or grade 4 transaminitis.	High-risk: (1) Diameter max ≥ 10 cm. (2) Dysfunction of vital organs, due to tumour compression. (3) Prospective life-threatening caused by tumour swelling. (4) Involvement of gastrointestinal tract with risks of bleeding and perforation.	Grade 3: Consistent with CARTOX.
	Grade 4: (1) Life-threatening hypotension. (2) Needing ventilator support. (3) Grade 4 organ toxicity (excluding transaminitis).		Grade 4: Consistent with CARTOX.
Management	Grade 1: (1) Antipyretics for the treatment of fever. (2) Maintenance intravenous fluids for hydration. (3) Management of constitutional symptoms and organ toxicities according to standard guidelines. (4) Consider tocilizumab or siltuximab for persistent and refractory fever. (5) Empiric antibiotic therapy if neutropenic.	Low-risk: Continuous clinical observation and supportive care as per standard guidelines.	Grade 1: (1) Anti-TNF-α agents as recommended for persistent and refractory fever. (2) Other management as consistent with CARTOX.
	Grade 2: (1) IV fluid bolus and supplemental oxygen. (2) Tocilizumab or siltuximab ± corticosteroids and supportive care, as recommended for the management of hypotension. (3) Manage fever and constitutional symptoms as in grade 1.		Grade 2: (1) Anti-IL-6 therapy ± anti-TNF-α agents as recommended. (2) Other managements are consistent with CARTOX.
	Grade 3: (1) IV fluid boluses and vasopressors as needed. (2) Transfer to ICU, and supplemental oxygen as needed. (3) Manage fever and constitutional symptoms as in grade 1. (4) Tocilizumab or siltuximab plus corticosteroids and supportive care, as recommended. (5) Symptomatic management of organ toxicities as per standard guidelines.	High-risk: (1) Continuous clinical observation and supportive care without adjacent organ compression symptoms. (2) Anti-TNF-α agents as needed in the presence of compression symptoms. (3) Local intervention (tracheotomy, drainage of serous effusion) as needed if clinically necessary. (4) Gut purge as recommended for large abdominal tumour lesions and gastrointestinal tract masses. (5) The prophylactic use of anti-TNF-α agents is considered.	Grade 3: (1) Anti-TNF-α agents as recommended if accompanied by L-CRS. (2) Plasma exchange as recommended. (3) Other managements are consistent with CARTOX.
	Grade 4: (1) IV fluids, anti-IL-6 therapy, vasopressors and methylprednisolone as recommended. (2) Mechanical ventilation. (3) Manage fever and constitutional symptoms as in grade 1. (4) Tocilizumab or siltuximab plus corticosteroids and supportive care, as recommended. (5) Symptomatic management of organ toxicities as per standard guidelines.		Grade 4: (1) Cyclophosphamide as required. (2) Other management as in grade 3.

*CARTOX* CAR T-cell-therapy-associated toxicity, *CRS* cytokine release syndrome, *B-NHL* B-cell non-Hodgkin lymphoma, *CAR* chimeric antigen receptor, *L-CRS* local cytokine release syndrome, *S-CRS* systemic cytokine release syndrome, *TNF-α* tumour necrosis factor-α, *IL-6* interleukin-6, *ICU* intensive care unit, *IV* intravenous injection

IL-6 plays an important role in the occurrence of S-CRS, and anti-IL-6 therapy has greatly improved the management of S-CRS. In addition, many clinical trials have shown that the use of tocilizumab does not impair therapeutic outcomes.^39,94^ Therefore, in various CRS management guidelines, tocilizumab is widely accepted and recommended across different CRS grades.^95^ However, some controversy remains regarding the use of anti-IL-6 agents. First, whether IL-6 blockade impairs the in vivo proliferation of CAR T cells is still debated, because IL-6 has been clearly proven to promote the proliferation of T cells.^96–98^ In addition, it has been reported that the serum IL-6 levels may rapidly rise after tocilizumab administration.^99^ According to our clinical observations, it is noteworthy that L-CRS can be aggravated by the use of tocilizumab. Although we are uncertain as to the mechanism behind this phenomenon, the rapid rise of IL-6 after tocilizumab administration may be an important factor. Therefore, when significant L-CRS occurs, tocilizumab is cautiously recommended for S-CRS management, and anti-TNF-α therapy might be a better choice. In our guiding principles, anti-TNF-α drugs are also recommended for patients with persistent fever, even if the S-CRS is below grade 2.

Two recently published articles demonstrated that monocytes and macrophages were the major contributors to CRS.^13,24^ Therefore, we believe that early blocking of the activation of monocytes and macrophages activation may be a reasonable strategy for CRS management. In addition to agents targeting TNF-α, blocking GM-CSF-mediated activation of monocyte and macrophage activation has been reported to effectively control the CAR T-cell-therapy-induced CRS response.^100,101^

## Summary and expectation

The in vivo kinetics of CAR T cells in B-NHL is different from that in ALL. As a result, CAR T-cell-treatment-related AEs in B-NHL exhibit some unique features, of which L-CRS is the most significant one. L-CRS is the earliest AE in CAR T-cell treatment for B-NHL, and we believe that timely management of L-CRS is beneficial to the prevention and control of serious S-CRS. However, the underlying mechanisms of L-CRS are not clear. Further active basic research will provide an important reference for the management of L-CRS. To guide clinical work and basic research, we tentatively proposed a new model to illustrate the progression of CRS in CAR T-cell treatment for B-NHL. In addition, we discussed how tumour burden and BMS interact to determine the progression of CRS. The recognition and management of L-CRS and S-CRS were also presented.

Some AEs are the result of differences in CAR T-cell manufacture,^102,103^ which should be avoided in the future because it is difficult to identify whether these AEs are universal. Although CAR T-cell products are individualized, the standardization and industrialization of CAR T-cell preparations may rise above some of the problems. At present, the number of CAR T-cell-treated B-NHL cases is still limited. The strict criteria for clinical trials may not enable a full understanding of real-world outcomes. More samples, especially from multiple institutions, need to be accumulated and studied to enrich our understanding of AEs. Only in this way can a more widely applicable clinical management consensus be formed.

Most of the aforementioned AEs were first recognized in the clinic and not observed in animal models, so the understanding based on current animal models is far from adequate. Clinical findings are in need of appropriate animal models to study mechanisms and developmental processes, to promote more appropriate AE recognition, management and the development of therapeutic drugs. At present, there is still a lack of drugs that can effectively manage L-CRS. Elucidation of the mechanisms of L-CRS will be helpful for the screening of drugs. Some emerging candidate drugs that inhibit inflammatory responses, such as adrenergic receptor inhibitors^104^ and JAK inhibitors,^105^ could be further explored in clinical trials.

## References

[1] Grupp SA (2013). Chimeric antigen receptor-modified T cells for acute lymphoid leukemia. N. Engl. J. Med..

[2] Maude SL (2014). Chimeric antigen receptor T cells for sustained remissions in leukemia. N. Engl. J. Med..

[3] Wang X (2016). Phase 1 studies of central memory-derived CD19 CAR T-cell therapy following autologous HSCT in patients with B-cell NHL. Blood.

[4] Schuster SJ (2017). Chimeric antigen receptor T cells in refractory B-cell lymphomas. N. Engl. J. Med..

[5] Kochenderfer JN (2017). Lymphoma remissions caused by anti-CD19 chimeric antigen receptor T cells are associated with high serum interleukin-15 levels. J. Clin. Oncol..

[6] Xu J (2019). Exploratory trial of a biepitopic CAR T-targeting B cell maturation antigen in relapsed/refractory multiple myeloma. Proc. Natl Acad. Sci. USA.

[7] Schuster SJ (2019). Tisagenlecleucel in adult relapsed or refractory diffuse large B-cell lymphoma. N. Engl. J. Med..

[8] Locke FL (2019). Long-term safety and activity of axicabtagene ciloleucel in refractory large B-cell lymphoma (ZUMA-1): a single-arm, multicentre, phase 1-2 trial. Lancet Oncol..

[9] Neelapu SS (2017). Axicabtagene ciloleucel CAR T-cell therapy in refractory large B-cell lymphoma. N. Engl. J. Med..

[10] Geyer MB (2019). First CAR to pass the road test: Tisagenlecleucel’s drive to FDA approval. Clin. Cancer Res..

[11] Axicabtagene ciloleucel (Yescarta) for B-cell lymphoma. *Med. Lett. Drugs Ther.* **60**, e122–e123. https://pubmed.ncbi.nlm.nih.gov/30036350/. (2018). 30036350

[12] Teachey DT (2016). Identification of predictive biomarkers for cytokine release syndrome after chimeric antigen receptor T-cell therapy for acute lymphoblastic leukemia. Cancer Discov..

[13] Giavridis T (2018). CAR T cell-induced cytokine release syndrome is mediated by macrophages and abated by IL-1 blockade. Nat. Med..

[14] Abramson JS (2018). Updated safety and long term clinical outcomes in TRANSCEND NHL 001, pivotal trial of lisocabtagene maraleucel (JCAR017) in R/R aggressive NHL. J. Clin. Oncol..

[15] Neelapu SS (2018). Chimeric antigen receptor T-cell therapy—assessment and management of toxicities. Nat. Rev. Clin. Oncol..

[16] Mahadeo KM (2019). Management guidelines for paediatric patients receiving chimeric antigen receptor T cell therapy. Nat. Rev. Clin. Oncol..

[17] Tanyi JL (2017). Possible compartmental cytokine release syndrome in a patient with recurrent ovarian cancer after treatment with mesothelin-targeted CAR-T cells. J. Immunother..

[18] Ding L (2018). Pleural cavity cytokine release syndrome in CD19-directed chimeric antigen receptor-modified T cell therapy: a case report. Medicine.

[19] Jin A (2019). Severe dyspnea caused by rapid enlargement of cervical lymph node in a relapsed/refractory B-cell lymphoma patient following chimeric antigen receptor T-cell therapy. Bone Marrow Transplant..

[20] Brudno JN, Kochenderfer JN (2016). Toxicities of chimeric antigen receptor T cells: recognition and management. Blood.

[21] Lee DW (2019). ASTCT consensus grading for cytokine release syndrome and neurologic toxicity associated with immune effector cells. Biol. Blood Marrow Transplant..

[22] Cazaux M (2019). Single-cell imaging of CAR T cell activity in vivo reveals extensive functional and anatomical heterogeneity. J. Exp. Med..

[23] Minn I (2019). Imaging CAR T cell therapy with PSMA-targeted positron emission tomography. Sci. Adv..

[24] Norelli M (2018). Monocyte-derived IL-1 and IL-6 are differentially required for cytokine-release syndrome and neurotoxicity due to CAR T cells. Nat. Med..

[25] Burger D, Dayer JM (2002). The role of human T-lymphocyte-monocyte contact in inflammation and tissue destruction. Arthritis Res..

[26] Liebana E, Aranaz A, Welsh M, Neill SD, Pollock JM (2000). In vitro T-cell activation of monocyte-derived macrophages by soluble messengers or cell-to-cell contact in bovine tuberculosis. Immunology.

[27] Burger D (2000). Cell contact-mediated signaling of monocytes by stimulated T cells: a major pathway for cytokine induction. Eur. Cytokine Netw..

[28] Sypek JP, Jacobson S, Vorys A, Wyler DJ (1993). Comparison of gamma interferon, tumor necrosis factor, and direct cell contact in activation of antimycobacterial defense in murine macrophages. Infect. Immun..

[29] Wagner DH, Stout RD, Suttles J (1994). Role of the CD40-CD40 ligand interaction in CD4+ T cell contact-dependent activation of monocyte interleukin-1 synthesis. Eur. J. Immunol..

[30] Nashleanas M, Scott P (2000). Activated T cells induce macrophages to produce NO and control Leishmania major in the absence of tumor necrosis factor receptor p55. Infect. Immun..

[31] McInnes IB, Leung BP, Sturrock RD, Field M, Liew FY (1997). Interleukin-15 mediates T cell-dependent regulation of tumor necrosis factor-alpha production in rheumatoid arthritis. Nat. Med..

[32] Avice MN, Sarfati M, Triebel F, Delespesse G, Demeure CE (1999). Lymphocyte activation gene-3, a MHC class II ligand expressed on activated T cells, stimulates TNF-alpha and IL-12 production by monocytes and dendritic cells. J. Immunol..

[33] Birkland TP, Sypek JP, Wyler DJ (1992). Soluble TNF and membrane TNF expressed on CD4+ T lymphocytes differ in their ability to activate macrophage antileishmanial defense. J. Leukoc. Biol..

[34] Parry SL, Sebbag M, Feldmann M, Brennan FM (1997). Contact with T cells modulates monocyte IL-10 production: role of T cell membrane TNF-alpha. J. Immunol..

[35] Wang Y (2014). Effective response and delayed toxicities of refractory advanced diffuse large B-cell lymphoma treated by CD20-directed chimeric antigen receptor-modified T cells. Clin. Immunol..

[36] Fried S (2019). Early and late hematologic toxicity following CD19 CAR-T cells. Bone Marrow Transplant..

[37] Lee DW (2015). T cells expressing CD19 chimeric antigen receptors for acute lymphoblastic leukaemia in children and young adults: a phase 1 dose-escalation trial. Lancet.

[38] Dholaria BR, Bachmeier CA, Locke F (2019). Mechanisms and management of chimeric antigen receptor T-cell therapy-related toxicities. BioDrugs.

[39] Singh N (2017). Monocyte lineage-derived IL-6 does not affect chimeric antigen receptor T-cell function. Cytotherapy.

[40] Hunter CA, Jones SA (2015). IL-6 as a keystone cytokine in health and disease. Nat. Immunol..

[41] Hay KA (2017). Kinetics and biomarkers of severe cytokine release syndrome after CD19 chimeric antigen receptor-modified T-cell therapy. Blood.

[42] Hay KA (2018). Cytokine release syndrome and neurotoxicity after CD19 chimeric antigen receptor-modified (CAR-) T cell therapy. Br. J. Haematol..

[43] Brentjens R, Yeh R, Bernal Y, Riviere I, Sadelain M (2010). Treatment of chronic lymphocytic leukemia with genetically targeted autologous T cells: case report of an unforeseen adverse event in a phase I clinical trial. Mol. Ther..

[44] Kochenderfer JN (2015). Chemotherapy-refractory diffuse large B-cell lymphoma and indolent B-cell malignancies can be effectively treated with autologous T cells expressing an anti-CD19 chimeric antigen receptor. J. Clin. Oncol..

[45] Mueller KT (2017). Cellular kinetics of CTL019 in relapsed/refractory B-cell acute lymphoblastic leukemia and chronic lymphocytic leukemia. Blood.

[46] Ali SA (2016). T cells expressing an anti-B-cell maturation antigen chimeric antigen receptor cause remissions of multiple myeloma. Blood.

[47] Teachey DT (2013). Cytokine release syndrome after blinatumomab treatment related to abnormal macrophage activation and ameliorated with cytokine-directed therapy. Blood.

[48] Menegazzi R, Cramer R, Patriarca P, Scheurich P, Dri P (1994). Evidence that tumor necrosis factor alpha (TNF)-induced activation of neutrophil respiratory burst on biologic surfaces is mediated by the p55 TNF receptor. Blood.

[49] Wright HL, Moots RJ, Bucknall RC, Edwards SW (2010). Neutrophil function in inflammation and inflammatory diseases. Rheumatology.

[50] McDonald PP, Russo MP, Ferrini S, Cassatella MA (1998). Interleukin-15 (IL-15) induces NF-kappaB activation and IL-8 production in human neutrophils. Blood.

[51] Turtle CJ (2016). Immunotherapy of non-Hodgkin’s lymphoma with a defined ratio of CD8+ and CD4+ CD19-specific chimeric antigen receptor-modified T cells. Sci. Transl. Med..

[52] Lee DW (2014). Current concepts in the diagnosis and management of cytokine release syndrome. Blood.

[53] Bonifant CL, Jackson HJ, Brentjens RJ, Curran KJ (2016). Toxicity and management in CAR T-cell therapy. Mol. Ther. Oncolytics.

[54] Schultz DR, Arnold PI (1990). Properties of four acute phase proteins: C-reactive protein, serum amyloid A protein, alpha 1-acid glycoprotein, and fibrinogen. Semin. Arthritis Rheum..

[55] Newick K, O’Brien S, Moon E, Albelda SM (2017). CAR T cell therapy for solid tumors. Annu. Rev. Med..

[56] Wang Z, Guo Y, Han W (2017). Current status and perspectives of chimeric antigen receptor modified T cells for cancer treatment. Protein Cell.

[57] Sun, L. et al. Inhibiting myeloid-derived suppressor cell trafficking enhances T cell immunotherapy. *JCI Insight* **4**. 10.1172/jci.insight.126853. (2019). 10.1172/jci.insight.126853 PMC6483637 30944253

[58] Porter DL (2015). Chimeric antigen receptor T cells persist and induce sustained remissions in relapsed refractory chronic lymphocytic leukemia. Sci. Transl. Med..

[59] Turtle CJ (2017). Durable molecular remissions in chronic lymphocytic leukemia treated with CD19-specific chimeric antigen receptor-modified T cells after failure of ibrutinib. J. Clin. Oncol..

[60] Santomasso BD (2018). Clinical and biological correlates of neurotoxicity associated with CAR T-cell therapy in patients with B-cell acute lymphoblastic leukemia. Cancer Discov..

[61] Yu S, Yi M, Qin S, Wu K (2019). Next generation chimeric antigen receptor T cells: safety strategies to overcome toxicity. Mol. Cancer.

[62] Yi M (2018). Gut microbiome modulates efficacy of immune checkpoint inhibitors. J. Hematol. Oncol..

[63] Brudno JN, Kochenderfer JN (2019). Recent advances in CAR T-cell toxicity: mechanisms, manifestations and management. Blood Rev..

[64] Locke, F. L., Go, W. Y. & Neelapu, S. S. Development and use of the anti-CD19 chimeric antigen receptor T-cell therapy axicabtagene ciloleucel in large B-cell lymphoma: a review. JAMA Oncol. 6, 281–290. https://jamanetwork.com/journals/jamaoncology/article-abstract/2754748. (2020). 10.1001/jamaoncol.2019.3869 PMC7859915 31697310

[65] Kochenderfer JN (2010). Eradication of B-lineage cells and regression of lymphoma in a patient treated with autologous T cells genetically engineered to recognize CD19. Blood.

[66] Brentjens RJ (2011). Safety and persistence of adoptively transferred autologous CD19-targeted T cells in patients with relapsed or chemotherapy refractory B-cell leukemias. Blood.

[67] Titov A (2018). The biological basis and clinical symptoms of CAR-T therapy-associated toxicites. Cell Death Dis..

[68] Neelapu SS (2019). CAR-T efficacy: is conditioning the key?. Blood.

[69] Turtle CJ (2016). CD19 CAR-T cells of defined CD4+:CD8+ composition in adult B cell ALL patients. J. Clin. Investig..

[70] Wallen H (2009). Fludarabine modulates immune response and extends in vivo survival of adoptively transferred CD8 T cells in patients with metastatic melanoma. PLoS ONE.

[71] Cordeiro A (2019). Late events after treatment with CD19-targeted chimeric antigen receptor modified T cells. Biol. Blood Marrow Transplant..

[72] Abramson JS (2019). Pivotal safety and efficacy results from transcend NHL 001, a multicenter phase 1 Study of lisocabtagene maraleucel (liso-cel) in relapsed/refractory (R/R) large B cell lymphomas. Blood.

[73] Hill JA (2018). Infectious complications of CD19-targeted chimeric antigen receptor-modified T-cell immunotherapy. Blood.

[74] Park JH (2018). Cytokine release syndrome grade as a predictive marker for infections in patients with relapsed or refractory B-cell acute lymphoblastic leukemia treated with chimeric antigen receptor T cells. Clin. Infect. Dis..

[75] Logue JM (2020). Immune reconstitution and associated infections following axicabtagene ciloleucel in relapsed or refractory large B-cell lymphoma. Haematologica.

[76] Gust J (2017). Endothelial activation and blood-brain barrier disruption in neurotoxicity after adoptive immunotherapy with CD19 CAR-T cells. Cancer Discov..

[77] Porter DL, Levine BL, Kalos M, Bagg A, June CH (2011). Chimeric antigen receptor-modified T cells in chronic lymphoid leukemia. N. Engl. J. Med..

[78] Frey N (2016). Optimizing chimeric antigen receptor (CAR) T cell therapy for adult patients with relapsed or refractory (r/r) acute lymphoblastic leukemia (ALL). J. Clin. Oncol..

[79] Hartmann J, Schussler-Lenz M, Bondanza A, Buchholz CJ (2017). Clinical development of CAR T cells-challenges and opportunities in translating innovative treatment concepts. EMBO Mol. Med..

[80] Dai, H., Wang, Y., Lu, X. & Han, W. Chimeric antigen receptors modified T-cells for cancer therapy. *J. Natl Cancer Inst*. **108**. 10.1093/jnci/djv439. (2016). 10.1093/jnci/djv439 PMC4948566 26819347

[81] Fesnak AD, June CH, Levine BL (2016). Engineered T cells: the promise and challenges of cancer immunotherapy. Nat. Rev. Cancer.

[82] Perez-Ruiz E (2019). Prophylactic TNF blockade uncouples efficacy and toxicity in dual CTLA-4 and PD-1 immunotherapy. Nature.

[83] Yi M (2019). Manipulating gut microbiota composition to enhance the therapeutic effect of cancer immunotherapy. Integr. Cancer Ther..

[84] Matson V (2018). The commensal microbiome is associated with anti-PD-1 efficacy in metastatic melanoma patients. Science.

[85] Routy B (2018). Gut microbiome influences efficacy of PD-1-based immunotherapy against epithelial tumors. Science.

[86] Gopalakrishnan V (2018). Gut microbiome modulates response to anti-PD-1 immunotherapy in melanoma patients. Science.

[87] Pinato DJ (2019). Antibiotic therapy and outcome from immune-checkpoint inhibitors. J. Immunother. Cancer.

[88] Pinato DJ (2019). Association of prior antibiotic treatment with survival and response to immune checkpoint inhibitor therapy in patients with cancer. JAMA Oncol..

[89] Dai H (2015). Tolerance and efficacy of autologous or donor-derived T cells expressing CD19 chimeric antigen receptors in adult B-ALL with extramedullary leukemia. Oncoimmunology.

[90] Ishii K (2016). Tocilizumab-refractory cytokine release syndrome (CRS) triggered by chimeric antigen receptor (CAR)-transduced T cells may have distinct cytokine profiles compared to typical CRS. Blood.

[91] Heczey A (2017). CAR T cells administered in combination with lymphodepletion and PD-1 inhibition to patients with neuroblastoma. Mol. Ther..

[92] Jain T (2019). Use of chimeric antigen receptor T cell therapy in clinical practice for relapsed/refractory aggressive B cell non-Hodgkin lymphoma: an expert panel opinion from the American Society for Transplantation and Cellular Therapy. Biol. Blood Marrow Transplant..

[93] Liu Y (2018). Hemofiltration successfully eliminates severe cytokine release syndrome following CD19 CAR-T-cell therapy. J. Immunother..

[94] Barrett DM, Singh N, Hofmann TJ, Gershenson Z, Grupp SA (2016). Interleukin 6 is not made by chimeric antigen receptor T cells and does not impact their function. Blood.

[95] Le RQ (2018). FDA approval summary: tocilizumab for treatment of chimeric antigen receptor T cell-induced severe or life-threatening cytokine release syndrome. Oncologist.

[96] Li B, Jones LL, Geiger TL (2018). IL-6 promotes T cell proliferation and expansion under inflammatory conditions in association with low-level RORgammat expression. J. Immunol..

[97] Takeda K (1998). Stat3 activation is responsible for IL-6-dependent T cell proliferation through preventing apoptosis: generation and characterization of T cell-specific Stat3-deficient mice. J. Immunol..

[98] Van Epps HL (2006). IL-6 drives T cell proliferation. J. Exp. Med..

[99] Nishimoto N (2008). Mechanisms and pathologic significances in increase in serum interleukin-6 (IL-6) and soluble IL-6 receptor after administration of an anti-IL-6 receptor antibody, tocilizumab, in patients with rheumatoid arthritis and Castleman disease. Blood.

[100] Sterner RM (2019). GM-CSF inhibition reduces cytokine release syndrome and neuroinflammation but enhances CAR-T cell function in xenografts. Blood.

[101] Sachdeva M, Duchateau P, Depil S, Poirot L, Valton J (2019). Granulocyte-macrophage colony-stimulating factor inactivation in CAR T-cells prevents monocyte-dependent release of key cytokine release syndrome mediators. J. Biol. Chem..

[102] Fraietta JA (2018). Disruption of TET2 promotes the therapeutic efficacy of CD19-targeted T cells. Nature.

[103] Ruella M (2018). Induction of resistance to chimeric antigen receptor T cell therapy by transduction of a single leukemic B cell. Nat. Med..

[104] Staedtke V (2018). Disruption of a self-amplifying catecholamine loop reduces cytokine release syndrome. Nature.

[105] Ahmed A (2019). Ruxolitinib in adult patients with secondary haemophagocytic lymphohistiocytosis: an open-label, single-centre, pilot trial. Lancet Haematol..

